# A Case Report of Post-Operative Jöd-Basedow Phenomennon Following Oral and IV Iodine Contrast Administration

**DOI:** 10.1155/2014/980283

**Published:** 2014-02-11

**Authors:** Maureen Higgs, Erroll Hull, Eugenio Lujan

**Affiliations:** Department of Anesthesia, Naval Medical Center, 34800 Bob Wilson Drive, San Diego, CA 92134, USA

## Abstract

This is a case of thyrotoxicosis, due to the Jöd-Basedow phenomenon following administration of oral and IV iodinated contrast in a patient with history of gastrointestinal stromal tumor (GIST) and small bowel obstruction. The patient developed atrial fibrillation and had an extended stay in the intensive care unit. Given the aging population with possible subclinical hyperthyroidism, multinodular goiter, and the rise in contrast administration for routine diagnostic studies, this case serves to raise awareness of the risks of “routine” tests administered to our aging patient population.

## 1. Introduction

Iodine is an essential requirement for thyroid hormone synthesis. For the thyroid gland to synthesize adequate amounts of thyroxine, it must take up approximately 52 mcg of iodide daily [1]. To obtain this amount of iodide, the recommended daily oral intake of iodine is 150 *μ*g for nonpregnant adults, with a tolerable upper level of 1100 *μ*g [2]. The average daily intake in the United States is 150–200 *μ*g, making the US an iodine replete population [3]. In many countries the daily iodine intake is much less, causing those populations to be iodine deficient.

Sources of iodine excess can include iodized salt, dietary supplementations, over-the-counter or prescription medications, and radiographic intravenous contrast media (ICM). A typical dose of ICM contains about 13,500 *μ*g of free iodide, of which 15–60 g of bound iodine may be liberated in the body. This infuses the body with an acute iodide load of 90 to several hundred thousand times the recommended daily intake [4].

When large amounts of iodine are encountered in subjects with normal thyroid function, sodium-iodide symporters (NIS) on follicular cells transport an increased amount of iodine intracellularly. The resultant increase in intrathyroidal iodine stores paradoxically blocks thyroid hormone organification. This TSH-independent autoregulatory block is called the Wolff-Chaikoff effect [1]. Within 24 hours of an exposure to iodine excess, the NIS expression is markedly decreased. Intrathyroidal iodine stores normalize, inducing resumption of normal thyroid function (escape from the Wolff-Chaikoff effect) [5].

Defective or absent autoregulatory mechanisms may lead to pathologic consequences of iodine excess. Failure to escape from the Wolff-Chaikoff effect may result in prolonged inhibition of thyroid hormone organification. This results in a rise in TSH and iodine induced hypothyroidism, which can be transitory or permanent in susceptible individuals [5]. Alternatively, some individuals, when exposed to an excess iodine load, will develop iodine-induced hyperthyroidism (IIH) or Jöd-Basedow disease. IIH is not a single etiologic entity. It may occur in patients with a variety of underlying thyroid diseases, the most common of which are Graves' disease and nodular goiter with thyroid autonomy [6]. Rarely, iodine excess may cause IIH in patients without underlying thyroid disease via acute onset thyroiditis [7].

Individuals who live in an iodine deficient area with underlying Graves' disease most commonly appear clinically euthyroid, as their hormone production is limited by the lack of dietary iodine. As more iodide is made available, hormone excretion increases, unmasking the Graves' disease with clinically evident thyrotoxicosis. Individuals with autonomous nodules present in a similar fashion. The autonomous nodules are active even in the absence of TSH, but held in check by a lack of available iodine. Once excess iodine is made available, sustained hormone synthesis occurs with eventual thyrotoxicosis [1].

We present the case of a seventy five year old male who experienced the Jöd-Basedow phenomenon after an abdominal computed tomography (CT) with contrast.

## 2. Consent for Publication

The patient and family were not contacted for permission to publish this case. Permission was not deemed necessary by the IRB. Public Affairs approval from the United States Navy was granted for publication of this case report.

## 3. Case Description

The patient was a 75-yr-old male with past medical history (PMHX) of gastrointestinal stromal tumor (GIST) involving the small bowel, sigmoid colon, and peritoneum, treated in 2008 with small bowel resection and sigmoid colectomy with L sided ostomy followed by long term Gleevac treatment. Additional PMHX includes prostate cancer, macrocytic anemia, pericardiotomy for pericardidtis 12 years prior, hypertension, hyperlipidemia, and restless leg syndrome.

The patient was admitted for partial small bowel obstruction. Initial management was medical with nasogastric (NG) tube decompression. By hospital day (HD) 2, the patient began advancing his diet, but on HD 3, he became hypotensive and developed a distended abdomen. He was subsequently taken to the CT scanner for CT with contrast (Oral 2.5% Omnipaque 1100 mL) of the abdomen and pelvis. He underwent an expoloratory laparotomy with partial small bowel resection and lysis of adhesions. He did well until post operative day (POD) 1, HD 4, when he became tachycardic, and was noted to be in new onset atrial fibrillation. Thyroid studies were drawn as part of the atrial fibrillation workup, and it was noted the patient was overtly hyperthyroid (Table 1). Diltiazem was started for rate control, and the patient was transferred to the surgical intesive care unit. An endocrine consult was placed. They noted the patient had never been informed of any past thyroid condition. He also had no compressive symptoms of the neck, no dysphagia, no pain in the region of the thyroid. On examination, patient did not demonstrate any lid lag, tremors, generalized warmth, or exophthalmos. The surgical intensive care attending did note a small, palpable, non-tender, hard nodule on the right inferior pole of the thyroid. It was noted, the patient had undergone a previous CT with contrast (Oral 2.5% Omnipaque 1100 mL and IV Isovue 120 mL) of the abdomen and pelvis two weeks earlier as an outpatient. A bedside ultrasound showed diffuse heterogeneity with numerous hypoechoic, subcentimeter nodules bilaterally, and a hyperechoic nodule in the inferior pole of the right lobe that measured 1 cm in diameter. Doppler flow surrounded the larger nodule as well as the smaller nodules bilaterally. Subsequently, the patient was started on methimazole. He had a formal ultrasound of the thyroid HD 10, which confirmed a 9 × 7 × 8 mm homogeneously, hyperechoic nodule in the posterior, inferior aspect of the right thyroid lobe. Color Doppler showed no internal vascularity. Bilaterally, multiple small, hypoechoic nodules showed vascularity. The thyroid lobes were normal in size and surrounding cervical tissues unremarkable.

Methimazole therapy improved the hyperthyroidism (Table 1), was tapered down over the next two months, and subsequently stopped.

## 4. Discussion

Iodine induced hyperthyroidism (IIH) or the Jöd-Basedow phenomenon, has been described in the literature, but in most cases, the patient experiences subclinical hyperthyroidism and remains euthyroid, or rarely develops overt hyperthyroidism after an average of 116 days [4, 6]. The biggest risk factors for manifesting overt hyperthyroidism after contrast are being elderly, having Grave's disease, living in an iodine depleted area, and multinodular goiter. This patient presented with an episode of acute thyrotoxicosis manifested as tachycardia and atrial fibrillation following two CT scans of the abdomen with contrast in a 13 day period. We believe that our patients nodule noted on both the bedside and formal ultrasound was an autonomous nodule, which when exposed to an iodine load, led to overt hyperthyroidism at an accelerated pace in comparison to other reports in the literature.

In some cases, a very rapid onset of thyrotoxicosis following ICM has been attributed to iodine induced thyroiditis [8]. In thyroiditis, there is follicular disruption with leakage of colloid from the thyroid. This leads to elevation of thyroxine and triiodothyronine, subsequently suppressing thyroid stimulating hormone. This condition is usually painful, and the patient typically presents with thyroidal pain [9]. The patient in our report did not have a tender, painful thyroid, making thyroiditis unlikely.

The most extreme cases of thyrotoxicosis following ICM are manifested as thyroid storm [10]. This condition typically has an abrupt onset, and is found to occur in patients with existing undertreated thyrotoxicosis [11]. With an estimated rate of occurrence of 0.0008%, thyroid storm precipitated by iodinated contrast media has rarely been described [5, 10]. Signs and symptoms of thyroid storm include fever, marked tachycardia, and arrhythmias, which may be accompanied by pulmonary edema or congestive heart failure, gastrointestinal dysfunction, and CNS involvement [10–12]. The point at which thyrotoxicosis becomes thyroid storm is subjective, and the diagnosis is clinical. Chen et al., [13] proposed a point system whereby a clinical score may be obtained to confirm or quantify the diagnosis. In practice, patients exhibiting significant symptoms of thyrotoxicosis should be assumed to have impending thyroid storm and undergo aggressive treatment, rather than waiting for a specific score threshold. In the case we present, the patient was treated with a calcium channel blocker and methimazole, after he developed an arrhythmia and tachycardia, with subsequent improvement in symptoms, making the diagnosis of thyroid storm unlikely.

Overall, this case of the Jöd-Basedow phenomenon, serves as a reminder that although rare, IIH can occur in an iodine replete country. Thousands of patients with subclinical hyperthyroidism, advanced age, and undiagnosed multinodular goiters undergo radiological studies with contrast on a daily basis. This patient serves as a clinical reminder to each provider, that our idodine replete, aging patient population is at risk for possible life threatening arrhythmias, increased morbidity, and worst case scenario death, from common diagnostic studies that we order on a daily basis.

## Figures and Tables

**Table 1:** Serial thyroid function tests after oral and IV contrast.

Test	TSH	FT4
(Ref. range)	(0.27–4.20) MIU/mL	(0.89–1.76) ng/dL
08/02/12*	0.16	3.21
08/23/12	0.78	1.97
09/20/12	2.12	1.51
10/01/12	3.74	1.41
10/12/12**	15.91	1.09
10/18/12	11.79	1.01
10/21/12	9.40	1.24
10/22/12	8.40	1.11
11/27/12	2.25	1.54

*Date methimazole therapy started.

**Date methimazole therapy stopped.
